# Antifungal prophylaxis of patients undergoing allogenetic hematopoietic stem cell transplantation in China: a multicenter prospective observational study

**DOI:** 10.1186/s13045-016-0305-y

**Published:** 2016-09-23

**Authors:** Lei Gao, Yuqian Sun, Fanyi Meng, Mingzhe Han, He Huang, Depei Wu, Li Yu, Hanyun Ren, Xiaojun Huang, Xi Zhang

**Affiliations:** 1 Xinqiao Hospital, Third Military Medical University, Chongqing, 400037 China; 2 Peking University Institute of Hematology, Peking University People’s Hospital, Beijing Key Laboratory of Hematopoietic Stem Cell Transplantation, Beijing, 100044 China; 3 Nanfang Hospital, Nanfang Medical University, Guangzhou, China; 4 Institute of Hematology and Blood Diseases Hospital, Chinese Academy of Medical Sciences, Tianjin, China; 5 The First Affiliated Hospital of Medical School of Zhejiang University, Hangzhou, China; 6 The First Affiliated Hospital of Soochow University, Suzhou, China; 7 Chinese PLA General Hospital (301 Hospital), Beijing, China; 8 The First Hospital of Peking University, Beijing, China

**Keywords:** Invasive fungal diseases, Allogenetic, Stem cell, Transplantation, Prophylaxis, China

## Abstract

**Background:**

Antifungal prophylaxis is currently regarded as the gold standard in situations with allo-genetic hematopoietic stem cell transplantation (allo-HSCT). However, the epidemiological information regarding prophylaxis of invasive fungal diseases (IFDs) is not clear in China.

**Methods:**

We report the first large-scale (1053 patients) observational study of the prophylaxis and management of IFDs among patients with allo-HSCT in China.

**Results:**

The incidence rates of IFD after primary antifungal prophylaxis (PAP), secondary antifungal prophylaxis (SAP), and non-prophylaxis were 22.7 vs. 38.6 vs. 68.6 %, respectively (*P* = 0.0000). The median time from transplantation to IFD was 45 days in PAP patients, 18 days in SAP patients, and 12 days in non-prophylaxis patients. *Aspergillus spp*. represents the most common type of fungal infection. Independent risk factors for IFD in allo-HSCT patients with PAP were age, having human leukocyte antigen (HLA)-haploidentical or matched unrelated donor, decreased albumin levels, and the use of itraconazole as the prophylactic antifungal agent. Among SAP transplant recipients, there was no significant risk factor for IFDs. The incidence rates of overall survival (OS) in the PAP, SAP, and no prophylaxis groups were 85.07, 78.80, and 74.82, respectively (*P* = 0.01).

**Conclusions:**

This observational study indicates that prophylaxis of IFD is helpful to reduce the incidence of IFDs and improve the OS of patients after allo-HSCT.

## Key points

This first large-scale observational study of invasive fungal disease (IFD) in China indicated that prophylaxis of IFD is helpful to improve the OS of patients after allo-genetic hematopoietic stem cell transplantation (allo-HSCT). For patients with a history of IFD, effective prevention of IFD is particularly important because they have higher incidence of IFD.

## Background

Although the control of bacterial infections in patients with hematological malignancies has been significantly improved with broad-spectrum antibiotics in the past decades, treating invasive fungal diseases (IFDs) is still a major problem in these patients, especially in patients undergoing allogenetic hematopoietic stem cell transplantation (allo-HSCT) [1–3]. Epidemiological data from the USA, China, and parts of Europe have shown that the incidence of IFDs in patients after allo-HSCT has increased dramatically in the past several years, substantially increasing the morbidity and mortality rates [4–9].

The diagnosis and treatment guidelines for IFDs, including some that are specific to HSCT patients, have been developed by academic societies in different world regions [10–12]. Notwithstanding the guidance and new forms of antifungal agents, treatment is often delayed because of nonspecific disease presentation and a lack of reliable diagnostic techniques, leading to poor clinical outcomes. Prophylaxis of IFDs is currently regarded as the gold standard in situations with allo-HSCT. Over the years, different scientific societies have established a series of recommendations for antifungal prophylaxis based on prospective studies performed with different drugs [13–17].

However, until recently, data on the prophylaxis of IFDs and real-world management of IFDs in patients with allo-HSCT have been gleaned primarily from single-center and retrospective studies in China. Here, we report the first large-scale observational study of the prophylaxis and management of IFDs among adults and children who have undergone allo-HSCT in China. Data came from the China Assessment of Antifungal Therapy in Hematological Disease (CAESAR) study.

## Methods

### Study design

The CAESAR study was a multicenter, prospective, observational study performed in 35 hematology centers across China, including two children’s hospitals. Subjects were consecutive patients of any age with a hematological malignancy who were hospitalized during the study period either after allogeneic or autologous HSCT or to receive intravenous chemotherapy. The overall study methods have been described previously [7, 18]. This observational study was conducted as a part of the CAESAR study and focused on the prophylaxis of IFDs in 1053 patients who underwent allo-HSCT in 31 HSCT centers.

All patients in each study center who were hospitalized and had undergone allo-HSCT between January 1, 2011 and October 30, 2011 were enrolled in this study. Data were collected by chart reviews and included the following parameters: demographic characteristics, antecedent hematologic disease, type of HSCT, conditioning regimen, IFD risk factors, clinical features suggestive of IFD, laboratory findings such as imaging, histopathology, and fungal cultures, treatment outcome of IFD, and mortality. Each IFD was categorized as proven, probable, or possible, according to the European Organization for Research and Treatment of Cancer/Invasive Fungal Infections Cooperative Group and the National Institute of Allergy and Infectious Diseases Mycoses Study Group (EORTC/MSG) 2008 criteria [19]. Patients were diagnosed as having suspected IFD if they had IFD risk factors; were observed to have symptoms, radiological abnormalities, or indirect microbiological evidence of fungal infection; and were treated empirically with antifungal agents but could not be diagnosed with proven, probable, or possible IFD according to the EORTC/MSG 2008 criteria [20]. Patients were followed for 6 months after the date of transplantation; the follow-up was completed on April 30, 2012. In all, 18 patients were lost to follow-up.

### Statistical analysis

In accordance with common practice and to maximize diagnostic accuracy, the incidence of IFD was mainly calculated based on proven and probable cases combined. The cumulative incidence was calculated as the incidence of proven plus probable IFD for the first 187 days after transplantation, divided by the number of cases at risk. Data were grouped according to primary antifungal prophylaxis (PAP) or secondary antifungal prophylaxis (SAP). PAP refers to patients without a history of fungal infection and need to be protected to avoid fungal infection after HSCT [21]. SAP is a rational strategy for patients with proven or probable IFD within 6 months before transplantation [22]. In particular, all allo-HSCT patients (*n* = 1045) in the CAESAR study were included in the analysis of overall survival (OS) for comparison of the OS differences in the PAP, SAP, and no prophylaxis groups. The characteristics of all of the allo-HSCT patients were described in the CAESAR study [7].

Statistics were primarily descriptive and were compared using analysis of variance, the Wilcoxon rank-sum test, or the chi-squared test, as appropriate. Risk factors for IFD were analyzed using univariate analysis followed by multivariate logistic regression. Kaplan-Meier analysis and the log-rank test were used to compare OS between different groups of patients. A two-sided *P* value of less than 0.05 was denoted as statistically significant. All statistical analyses were performed with SAS software version 9.1 (SAS, Cary, NC, USA).

## Results

### Patient characteristics and antifungal prophylaxis regimen

A total of 1053 patients who underwent allo-HSCT were enrolled from 31 HSCT centers across China. Their baseline demographic and clinical characteristics at the time of admission for transplantation are shown in Table 1. Among them, 906 patients received prophylactic antifungal treatment before or after transplantation. Previous IFDs were noted in 88 patients (8.4 %, 88/1053), including 7 with proven IFDs and 81 with probable IFDs.

**Table 1:** Patient characteristics and antifungal prophylaxis regimen

Characteristic	PAP (*N* = 818)	SAP (*N* = 88)	Non-antifungal prophylaxis (*n* = 147)
Age, mean (range/%)/case	28.5 (1–63)	32.2 2–58)	29.6 (4–61)
0–6	33 (4 %)	3 (3.4 %)	4 (2.7 %)
>6–16	129 (15.8 %)	10 (11.4 %)	20 (13.6 %)
>16–40	488 (59.7 %)	49 (55.7 %)	85 (57.8 %)
>40–65	168 (20.5 %)	26 (29.5 %)	38 (25.9 %)
Gender			
Male	505 (61.7 %)	51 (58.0 %)	87 (59.2 %)
Female	313 (38.3 %)	37 (42.0 %)	60 (40.8 %)
E-COG			
0	227 (27.8 %)	18 (20.5 %)	48 (32.7 %)
1	438 (53.5 %)	54 (61.4 %)	74 (50.3 %)
2	89 (10.9 %)	11 (12.5 %)	17 (11.6 %)
3	51 (6.2 %)	5 (5.7 %)	7 (4.8 %)
4	13 (1.6 %)	–	1 (0.7 %)
Underlying disease			
Acute myeloid leukemia	289 (35.3 %)	44 (50.0 %)	43 (29.3 %)
Acute lymphocytic leukemia	241 (29.5 %)	32 (36.4 %)	37 (25.2 %)
Chronic myeloid leukemia	100 (12.2 %)	3 (3.4 %)	23 (15.6 %)
Aplastic anemia	69 (8.4 %)	–	13 (8.8 %)
Myelodysplastic syndrome	62 (7.6 %)	2 (2.3 %)	16 (10.9 %)
Non-Hodgkin’s lymphoma	23 (2.8 %)	4 (4.5 %)	4 (2.7 %)
Chronic lymphocytic leukemia	4 (0.5 %)	–	–
Multiple myeloma	2 (0.2 %)	–	1 (0.7 %)
Hodgkin’s disease	2 (0.2 %)	–	3 (2.0 %)
Myeloprolififerative neoplasms	2 (0.2 %)	–	–
Solid tumor	1 (0.1 %)	–	–
Hereditary and metabolic disorders	9 (1.1 %)	–	–
Paroxysmal nocturnal hemoglobinuria	1 (0.1 %)	–	1 (0.7 %)
Others^a^	13 (1.6 %)	3 (3.4 %)	6 (4.1 %)
Transplantation type			
HLA-matched related (sibling)	335 (41.0 %)	35 (39.8 %)	77 (52.4 %)
Haploidentical	269 (32.9 %)	26 (29.5 %)	35 (23.8 %)
Unrelated	213 (26.0 %)	27 (30.7 %)	35 (23.8 %)
Source of stem cells			
PB	417 (51.0 %)	67 (76.1 %)	105 (71.4 %)
BM + PB	348 (42.5 %)	19 (21.6 %)	20 (13.6 %)
BM	33 (4.0 %)	–	7 (4.8 %)
CB	15 (1.8 %)	1 (1.1 %)	3 (2.0 %)
BM + CB	3 (0.4 %)	–	–
BM + PB + CB	2 (0.2 %)	1 (1.1 %)	8 (5.4 %)
Conditioning regimen			
Myeloablative			
Yes	729 (89.1 %)	83 (94.3 %)	124 (84.4 %)
No	89 (10.9 %)	5 (5.7 %)	23 (15.6 %)
Total body radiotherapy			
Yes	122 (14.9 %)	23 (26.1 %)	13 (8.8 %)
No	696 (85.1 %)	65 (73.9 %)	134 (91.2 %)
Antithymocyte globulin			
Yes	516 (63.1 %)	52 (59.1 %)	64 (43.5 %)
No	302 (36.9 %)	36 (40.9 %)	83 (56.5 %)
Concomitant disease	137 (16.7 %)	22 (25.0 %)	23 (15.6 %)
Cardiovascular disease	24 (2.9 %)	2 (2.3 %)	4 (2.7 %)
Diabetes	24 (2.9 %)	2 (2.3 %)	6 (4.1 %)
Viral hepatitis	22 (2.7 %)	9 (10.2 %)	4 (2.7 %)
CMV infection	1 (0.1 %)	2 (2.3 %)	1 (0.7 %)
Tuberculosis	14 (1.7 %)	3 (3.4 %)	2 (1.4 %)
Autoimmune diseases	9 (1.1 %)	1 (1.1 %)	–
Others^b^	61 (7.5 %)	5 (5.7 %)	8 (5.4 %)
aGVHD			
I–II aGVHD	257 (31.4 %)	27 (30.7 %)	40 (27.2 %)
III–IV aGVHD	57 (7.0 %)	7 (8.0 %)	15 (10.2 %)
cGVHD			
Limited	57 (7.0 %)	9 (10.3 %)	10 (6.85)
Extensive	24 (2.9 %)	2 (2.3 %)	4 (2.7 %)
Drugs for IFD prophylaxis			
Single drug	667 (81.5 %)	69 (78.4 %)	–
Fluconazole	429 (64.3 %)	8 (11.6 %)	–
Itraconazole	103 (15.4 %)	17 (24.6 %)	–
Voriconazole	71 (10.6 %)	32 (46.4 %)	–
Caspofungin	4 (0.6 %)	6 (8.7 %)	–
Amphotericin B	2 (0.3 %)	1 (1.4 %)	–
Drug combination	151 (18.5 %)	19 (21.6 %)	–
Route of administration			
Oral	466 (57.0 %)	29 (33.0 %)	–
Intravenous	214 (26.2 %)	37 (42.0 %)	–
Oral + intravenous	138 (16.9 %)	22 (25.0 %)	–
Time of prophylaxis before HSCT mean (SD), days	10.8 (6.57)	12.0 (6.43)	–

*E-COG* Eastern Cooperative Oncology Group, *PAP* primary antifungal prophylaxis, *SAP* secondary antifungal prophylaxis, *BM* bone marrow, *PB* peripheral blood, *CB* cord blood, *CMV* cytomegalovirus, *aGVHD* acute graft versus host disease, *cGVHD* chronic graft versus host disease, *IFD* invasive fungal diseases, *HSCT* hematopoietic stem cell transplantation

^a^Others include chronic myelomonocytic leukemia (*n* = 3), T lymphoblastic lymphoma leukemia (*n* = 2), mixed-lineage leukemia (*n* = 10), aggressive NK cell leukemia (*n* = 3), myeloid sarcoma (*n* = 1), plasmablasticlymphoma (*n* = 3)

^b^Others include infectious disease (*n* = 18), digestive system diseases (*n* = 10), cholecystolithiasis (*n* = 7), mouth disease (*n* = 8), urological diseases (*n* = 6), intestinal diseases (*n* = 7), endocrine diseases (*n* = 7), surgery (*n* = 5), cerebrovascular disease (*n* = 4), tumor (*n* = 2)

The average duration of antifungal prophylaxis before HSCT was 10.9 ± 6.56 days (10.8 ± 6.57 days in the PAP group and 12.0 ± 6.43 days in the SAP group). Single antifungal agents were the most common prophylaxis regimen (667/818 in the PAP group and 69/88 in the SAP group). Fluconazole (429/818, 64.9 %) and voriconazole (32/88, 46.4 %) were the most widely used agents for PAP and SAP, respectively (Table 1). All antifungal agents were given in accordance with the recommended doses and schedule.

### Efficacy of PAP and SAP on the prophylaxis of IFD occurrence

In the 1053 patients, 321 (30.5 %) patients were identified as having an IFD, including 13 (1.2 %) with proven IFD, 81 (7.7 %) with probable IFD, and 227 (21.6 %) with possible IFD. The incidence rates of proven, probable, and possible IFD in the PAP, SAP, and non-prophylaxis groups were 0.7 vs. 0.0 vs. 4.8 %, 7.0 vs. 11.4 vs. 9.5 %, and 15.0 vs. 27.3 vs. 54.4 %, respectively (Table 2). The median time from transplantation to IFD was 45 days in the PAP group (interquartile range (IQR), 16 to 75), 18 days in the SAP group (IQR, 7 to 26), and 12 days in the non-prophylaxis group (IQR, 0 to 93), respectively.

**Table 2:** The recurrence rates of IFD in patients from the PAP, SAP, and non-prophylaxis groups

Characteristic	PAP (*N* = 818)	SAP (*N* = 88)	Non-prophylaxis (*n* = 147)	*P* value
IFD patients	186 (22.7 %)	34 (38.6 %)	101 (68.7 %)	0.0000
Proven	6 (0.7 %)	0 (0.0 %)	7 (4.8 %)	
Probable	57 (7.0 %)	10 (11.4 %)	14 (9.5 %)	
Possible	123 (15.0 %)	24 (27.3 %)	80 (54.4 %)	
Patients without IFD	632 (77.3 %)	54 (61.4 %)	46 (31.3 %)	

### Characteristics of patients with IFD occurrence

Thirteen patients were diagnosed with proven IFD (6 in the PAP group and 7 in the non-prophylaxis group), and 81 patients were diagnosed with probable IFD (57 in the PAP group, 10 in the SAP group, and 14 in the non-prophylaxis group). Among the 94 patients with IFD, 56 were male and 38 were female. The underlying diseases were acute myeloid leukemia (*n* = 32), acute lymphocytic leukemia (*n* = 32), myelodysplastic syndrome (*n* = 10), chronic myeloid leukemia (*n* = 8), non-Hodgkin’s lymphoma (*n* = 5), aplastic anemia (*n* = 5), and others (*n* = 2). Twenty patients received human leukocyte antigen (HLA)-matched sibling HSCT, 42 patients received HLA-haploidentical HSCT, and 32 patients received HLA-matched unrelated HSCT.

Detailed data regarding the distribution of fungal pathogens and the methods of microbiological diagnoses of IFDs are shown in Table 3. Of the 89 etiological pathogens identified in patients with proven or probable IFDs, 62 (69.7 %) were *Aspergillus*, including 19 that were exclusively diagnosed with two positive galactomannan tests. Unspecified *Aspergillus* was the most common mold (31 cases), followed by A*spergillus flavus* (5 cases), and A*spergillus fumigatus* (2 case) isolates.

**Table 3:** Identified etiological pathogens in IFD cases

Fungal species	Overall population	PAP	SAP	Non-prophylaxis
Overall	89	55	7	27
*Candida spp*.	27	14	3	10
Unclassified *Candida*	8	5	0	3
*Candida tropicalis*	3	2	0	1
*Candida albicans*	3	2	0	1
*Candida parapsilosis*	3	2	0	1
*Candida krusei*	3	1	1	1
*Candida glabrata*	1	1	0	0
*Candida albicans*	5	1	2	2
Not specified^a^	1	0	0	1
*Aspergillus spp*.	62	41	4	17
Positive GM tests only	19	15	1	3
*Aspergillus flavus*	5	3	0	2
*Aspergillus versicolor*	1	1	0	0
*Aspergillus fumigatus*	2	2	0	0
*Aspergillus albicans*	1	0	0	1
*Mucor*	1	0	0	1
*Cryptococcus*	1	1	0	0
*Pityrosporion ovale*	1	1	0	0
Not specified^b^	31	18	3	10

^a^Including cases with yeasts identified in tissue but negative cultures

^b^Including cases with hyphae identified in tissue but negative cultures

The infection sites of all categories of IFD were also analyzed. Of all the 54 infection sites identified in patients with IFDs, the most common location of infection was the lower respiratory tract (64.8 %, 35/54), followed by blood stream infections only (13.0 %, 7/54), central nervous system (5.6 %, 3/54), spleen (1.9 %, 1/54), and three other sites (14.8 %, 8/54). Further analysis showed that *Aspergillus* is the main pathogen of lower respiratory tract fungal infection (85.7 %, 30/35). In other infection sites, there was no significant difference in the infection rate between *Aspergillus* and *Candida*.

### Risk factors for proven and probable IFD

The risk factors for proven and probable IFDs among PAP and SAP transplant recipients are presented in Tables 4 and 5. Univariate analyses revealed that HLA-haploidentical HSCT, HLA-matched unrelated HSCT, the use of antithymocyte globulin, prolonged profound neutropenia (>14 days), renal impairment, decreased albumin levels, and Epstein-Barr virus and cytomegalovirus viremia were independent risk factors for proven and probable IFDs in allo-HSCT patients with PAP (*P* < 0.05). Among the allo-HSCT patients with SAP, the use of antithymocyte globulin was the only obviously significant risk factor (*P* = 0.043).

**Table 4:** Risk factors for proven/probable IFD among allo-HSCT patients in the PAP group

Factor	Univariate analysis			Multivariate analysis		
	Patients with proven/probable IFD (*n*/*N*)	Incidence of proven/probable IFD (%)	Comparison (*P* value)	SE	OR (95 % CI)	Comparison (*P* value)
Age						
≤18 years old	9/182	4.95	0.15	0.44	2.78 (1.18–6.55)	*0.02*
>18 years old	54/636	8.49				
Transplantation type						
HLA-matched related (sibling)	10/335	2.99	*0.0001*			
Haploidentical	31/269	11.52		0.65	6.08 (1.69–21.86)	*0.01*
Unrelated	22/213	10.33		0.60	8.07 (2.50–26.10)	*0.00*
Antithymocyte globulin						
Yes	49/516	4.64	*0.01*	0.57	0.40 (0.13–1.24)	0.11
No	14/302	9.50				
Glucocorticoids^a^						
Yes	58/678	8.55	0.05	0.58	1.37 (0.44–4.23)	0.58
No	5/140	3.57				
GVHD^b^						
Non-GVHD	31/428	7.24	0.15	0.40	1.63 (0.74–3.58)	0.22
aGVHD I–II	16/230	6.96				
aGVHD III–IV	8/52	15.38				
cGVHD local	2/60	3.33				
cGVHD extensive	3/27	11.11				
Prolonged, profound neutropenia						
ANC > 500/mm^3^	1/14	7.14	*0.0006*			
ANC < 500/mm^3^, <10 days	6/142	4.23		1.22	0.40 (0.04–4.33)	0.45
ANC < 500/mm^3^, 10–14 days	4/176	2.27		1.28	0.14 (0.01–1.73)	0.13
ANC < 500/mm^3^, >14 days	52/486	10.70		1.16	0.99 (0.10–9.60)	0.99
EBV viremia^c^						
Yes	10/62	16.13	*0.03*	0.44	1.35 (0.58–3.17)	0.49
No	43/646	6.66				
Untested	10/110	9.09				
CMV viremia^c^						
Yes	33/267	12.36	*0.004*	0.33	1.18 (0.61–2.26)	0.63
No	29/528	5.49				
Untested	1/23	4.35				
Renal impairment						
Yes	10/60	16.67	*0.019*	0.43	1.38 (0.59–3.24)	0.46
No	53/758	6.99				
Decreased albumin						
Yes	39/317	12.30	*0.0001*	0.31	1.98 (1.08–3.62)	*0.03*
No	24/501	4.79				
Time of IFD prophylaxis						
<35 days	33/396	8.33	0.60	0.30	1.33 (0.74–2.39)	0.35
≥35 days	30/416	7.21				
Drugs for IFD prophylaxis						
Fluconazole/fluconazole + itraconazole^e^	39/498	7.83	0.17	0.46	1.76 (0.72–4.31)	0.21
Itraconazole^e^	14/103	13.59		0.53	3.14 (1.11–8.86)	*0.03*
Voriconazole^e^	2/71	2.82		0.85	0.95 (0.18–5.01)	0.95
Other^d^	8/144	5.56				

*ANC* absolute neutrophil count, *EBV* Epstein-Barr virus

^a^Including dexamethasone, methylprednisolone, prednisone, and hydrocortisone

^b^By multivariate analysis, OR is for cGVHD extensive/aGVHD III and IV degree versus cGVHD local/aGVHD I and II degree/non-GVHD

^c^By multivariate analysis, OR is for with EBV viremia versus without EBV viremia or untested/CMV viremia versus without CMV viremia or untested

^d^Including caspofungin, micafungin, amphotericin B, fluconazole + caspofungin, fluconazole + micafungin, fluconazole + voriconazole, itraconazole + micafungin, voriconazole + caspofungin, and fluconazole + caspofungin + voriconazole

^e^Compared with others

The italicized data reflected significant difference

**Table 5:** Risk factors for proven/probable IFD among allo-HSCT patients in SAP group

Factor	Univariate analysis			Muitivariate analysis		
	Patients with proven/probable IFD (*n*/*N*)	Incidence of proven/probable IFD (%)	Comparison (*P* value)	SE	OR (95 % CI)	Comparison (*P* value)
Transplantation type						
HLA-matched related (sibling)	1/35	2.86	0.08			
Haploidentical	5/26	19.23		1.77	2.48 (0.08–79.46)	0.61
Unrelated	4/27	14.81		1.77	2.24 (0.07–72.11)	0.65
Antithymocyte globulin						
Yes	9/52	17.31	*0.04*	1.68	2.49 (0.09–67.66)	0.59
No	1/36	2.78				
Prolonged, profound neutropenia						
ANC > 500/mm^3^	0/1	0.00	0.23	1.13	5.90 (0.65–53.89)	0.12
ANC < 500/mm^3^, <10 days	1/18	5.56				
ANC < 500/mm^3^, 10–14 days	0/17	0.00				
ANC < 500/mm^3^, >14 days	9/52	17.31				
Liver impairment						
Yes	5/24	20.83	0.12	0.74	2.07 (0.49–8.79)	0.33
No	5/64	7.81				
Time of IFD prophylaxis						
<35 days	5/40	12.50	1.00	0.76	1.10 (0.25–4.86)	0.90
≥35 days	5/48	10.42				
Drugs for IFD prophylaxis						
Fluconazole/itraconazole/	3/27	11.11	1.00	0.96	0.87 (0.13–5.64)	0.88
Fluconazole + itraconazole^b^						
Voriconazole^b^	4/32	12.50		0.91	1.50 (0.25–8.96)	0.66
Others^a^	3/29	10.34				

^a^Including voriconazole + caspofungin and fluconazole + caspofungin + voriconazole

^b^Compared with others

The italicized data reflected significant difference

Multivariate analyses demonstrated that independent risk factors for proven and probable IFDs in allo-HSCT patients with PAP were age >18 years (odds ratio (OR), 2.78; 95 % confidence interval (CI), 1.18–6.55; *P* < 0.05), having an HLA-matched unrelated donor (OR, 8.07; 95 % CI, 2.50–26.10; *P* < 0 .01), having an HLA-haploidentical donor (OR, 6.08; 95 % CI, 1.69–21.86; *P* < 0.01), decreased albumin levels (OR, 1.98; 95 % CI, 1.08–3.62; *P* < 0.05), and the use of itraconazole as the antifungal prophylactic agent (OR, 3.41; 95 % CI, 1.11–8.86; *P* < 0 .05) (Table 4). Among the SAP transplant recipients, there was no significant risk factor for proven or probable IFD.

### Overall antifungal therapy

A total of 321 IFD patients were provided therapeutic antifungal treatment. A single antifungal agent was used for treatment in 116 (36.1 %) patients; 144 (44.9 %) patients required two agents, and 61 (19.0 %) patients required three or more agents during the course of their treatment. Among the PAP transplant recipients, itraconazole (34.4 %, 64/186 in all-category IFD; 46.1 %, 29/63 in proven or probable IFD) was the most popular agent for the initial therapy. Among the SAP and non-prophylaxis transplant recipients, voriconazole (36.3 %, 49/135 in all-category IFD; 25.8 %, 8/31 in proven or probable IFD) was the most common agent for the initial therapy. Whether the patients in PAP, SAP, or non-prophylaxis groups, voriconazole as a single agent or in combination with other agents (35.8 %, 115/321) was the most popular agent for antifungal treatment.

The median overall treatment duration in patients who started and completed antifungal treatment while hospitalized was 36 days (IQR, 21–65) for PAP transplant recipients, 39 days (IQR, 24–58) for SAP transplant recipients, and 35 days (IQR, 20–67) for non-prophylaxis transplant recipients. A total of 175 patients (54.5 %, 94 in the PAP group, 20 in the SAP group, and 61 in the non-prophylaxis group) continued treatment after leaving the hospital.

### Outcomes including mortality

**Fig. 1:**
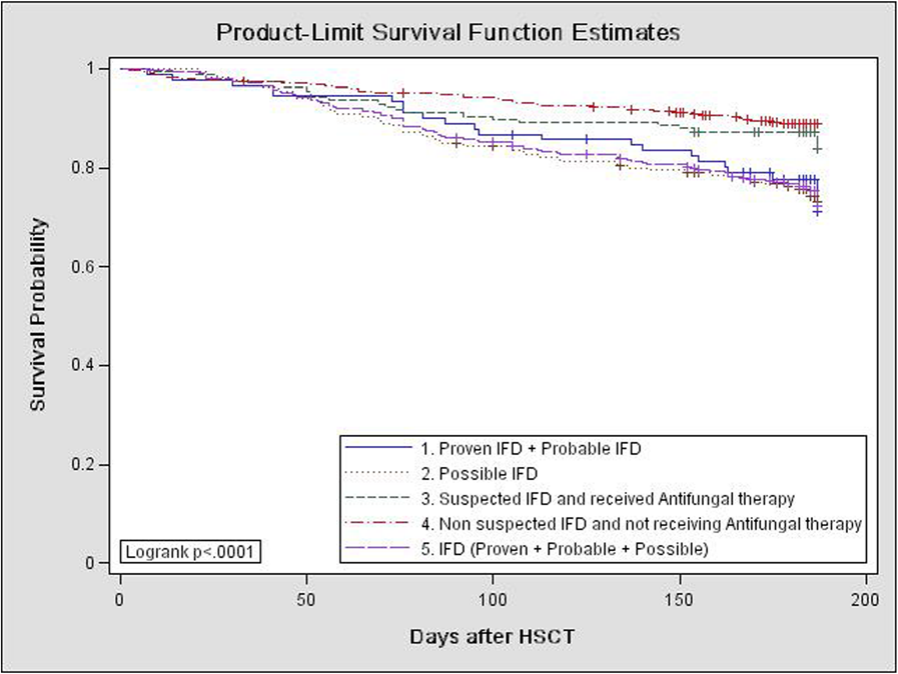
Overall survival of patients with proven/probable IFD, possible IFD, or suspected IFD and who received antifungal therapy; those with no suspected IFD and who did not receive antifungal therapy; and those without any IFD (proven, probable, or possible)

**Fig. 2:**
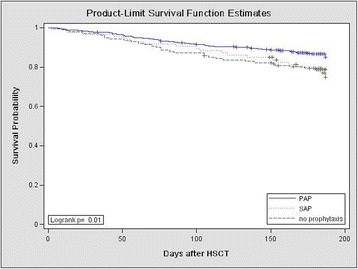
Overall survival of allo-HSCT patients receiving PAP, SAP, or no prophylaxis

At the end of follow-up, 171 patients had died, resulting in an overall mortality rate of 16.2 % (171/1053). Compared with the overall study population, the mortality rate was markedly higher in patients with proven (5/13; 38.5 %), probable (19/81; 23.5 %), or possible (58/227; 25.6 %) IFDs. Furthermore, the mortality rate among patients treated for suspected IFD despite failing to meet the EORTC/MSG diagnostic criteria [16] (25/166; 15.1 %) was higher than that among patients who were not suspected of having IFD and, therefore, not treated with antifungal agents (64/566; 11.3 %) (Fig. 1, *P* < 0.001). In addition, we compared the incidence of OS in the PAP, SAP, and no prophylaxis groups. There was a significant difference among the three groups (85.07 vs. 78.80 vs. 74.82 %, respectively, Fig. 2, *P* = 0.01).

## Discussion

Invasive infections remain major infectious threats to patients undergoing allo-HSCT and are associated with high fatality rates [23–28]. The diagnosis of IFDs continues to be difficult to establish because they do not manifest with specific clinical or radiographic signs or symptoms [29]. In the recent years, there has been a concerted effort to identify alternative procedures for the future diagnosis of fungus. They include the targeting of fungal antigens by enzyme-linked immunosorbent assay (ELISA) or lateral flow devices (LFDs) [30], detection of siderophores [31], and amplification of fungal nucleic acids from tissue and body fluids [32] as well as application of matrix-assisted laser desorption ionization TOF mass spectrometry (MALDI-TOF MS) [33]. Each of the above methods has advantages and disadvantages, and not all of which have entered clinical practice [34]. The difficulty in obtaining a timely diagnosis as well as the high morbidity and mortality rates associated with IFDs provide a rationale for antifungal prophylaxis in patients undergoing allo-HSCT. Nonetheless, antifungal prophylaxis remains a topic of some controversy, with no clear consensus among different centers [35–37].

The CAESAR study is the first population-based and the largest prospective observational study of the incidences of IFDs in patients receiving HSCT [7]. This observational study was conducted as a part of the CAESAR study and focused on the prophylaxis of IFDs in patients who underwent allo-HSCT. In the present analysis of adults and children at risk for IFD due to allo-HSCT, non-prophylaxis transplant recipients had a higher incidence of proven/probable/possible IFD than PAP and SAP transplant recipients at 6 months (68.6 vs. 22.7 and 38.6 %, *P* = 0.0000). This result was in accord with the classic randomized clinical trials on PAP in HSCT recipients and patients with hematological malignancies [38–40]. Furthermore, the OS was significantly different among PAP, SAP, and no prophylaxis patients. It was revealed that antifungal prophylaxis was helpful to reduce the incidence of IFD and improve the survival of patients after transplantation.

The most recently published European guidelines pointed out that SAP should be administered to patients with a previous IFD to prevent recurrence of the previous IFD or onset of a new IFD during a new at-risk phase, mainly referring to a prolonged neutropenic period induced by chemotherapy or a phase of severe immunosuppression after allo-HSCT [16]. Several studies have reported success rates for SAP, which was proven to be effective in preventing IFD recurrence [41, 42]. In the study, the cumulative incidence of IFD increased particularly rapidly during the first month after transplantation in the SAP group and non-prophylaxis group, suggesting that this is a high-risk period during which health care providers should pay particularly close attention to signs of emerging IFD in SAP and non-prophylaxis patients. On the contrary, the median time of IFD occurrence in the PAP group was 45 days after HSCT. This finding indicated that patients who had a previous IFD and had no antifungal prophylaxis were more likely to experience a breakthrough fungal infection in the early stage after transplantation.

As 22.7 % of patients treated with PAP and 38.6 % of patients treated with SAP in the study went on to develop proven, probable, or possible IFD, there remains an unmet need for education about the appropriate timing and choice of antifungal agent for prophylaxis in China. Among PAP transplant recipients, the most commonly prescribed prophylactic agents were fluconazole and itraconazole, which may be less effective than posaconazole [43]. The use of itraconazole as the antifungal prophylactic agent also proved the independent risk factor for IFD occurrence in our study. An update to the cost-effectiveness of posaconazole vs. fluconazole or itraconazole in the prevention of IFD among neutropenic patients in the USA has shown that posaconazole is a cost-effective alternative to fluconazole or itraconazole in the prevention of IFD among neutropenic patients [44]. Among SAP transplant recipients, there was no significant risk factor for proven or probable IFD. The characteristics of the observational study and the small sample size of the patients treated with SAP may be the main reasons. Prospective and randomized studies assessing the risk factors for SAP are needed in the future.

The epidemiological characteristics of IFD continue to evolve in transplant patients. A major contributor is the widespread use of azole prophylaxis since the early 1990s, which results in less candidiasis but more frequent mold infections in hematologic malignancies [25, 45]. In most [46–48] but not all [49] cases, *Aspergillus spp*. represent the most frequent cause of fungal-related morbidity in patients with HSCT. In our study, *Aspergillus spp*. also predominated in culture-proven or histologically proven pathogens and was more than two times as common as *Candida spp*. The mortality among patients who developed IFD (24.1 %) was almost double than that in the overall population of HSCT patients (15.0 %), and regression analysis confirmed that the development of IFD is a significant independent risk factor for death, highlighting the grave prognosis of those with IFD and the need for a timely diagnosis and prompt treatment. The incidence rates of invasive aspergillosis, however, need to be interpreted with caution as establishing this diagnosis often requires invasive procedures that are difficult to perform in severely ill patients. The low rate of proven/probable IFD in the present study suggests the need to improve diagnostic techniques to treat IFD as early and accurately as possible. Under the existing conditions, in order to reduce the IFD-related mortality of allo-HSCT patients, preemptive antifungal therapy should be given to patients with possible or suspected IFD.

The current study has several limitations. The main limitation was its observational nature. Confounding factors cannot be controlled effectively in observational studies, frequently due to biased selection of patients or treatment protocols. In the analysis of mortality by antifungal therapy, the uncontrolled study design means that no firm conclusions can be drawn regarding the relative effect of different treatments. Another limitation was the relatively short duration. The fact that the overall treatment duration was not recorded in patients who were still taking antifungal treatment after discharge from the hospital further limited data analysis. A longer follow-up would have allowed a more comprehensive analysis of patients who developed IFD after discharge. Procedures for diagnosis, prophylaxis, and treatment of IFD were not prespecified in the protocol but were performed according to usual practice and local clinical guidelines [19]. Furthermore, in some centers, diagnostic procedures were conducted only among patients with suspected clinical signs or symptoms of IFD. The observational study also meant that the diagnosis of specific infections, although guided by EORTC/MSG 2008 criteria, was limited to the data collected according to local hospitals’ usual practice; there was no mandatory requirement for diagnostic microbiological testing or the use of a centralized laboratory to validate the results.

## Conclusions

In conclusion, the results of the present observational study indicate that prophylaxis of IFD among patients receiving allo-HSCT for hematological malignancy in China is broadly in line with the recommended practice and is helpful to reduce the incidence of IFD and improve the OS of patients after transplantation. For patients with a history of IFD, active and effective prevention of fungal infections is particularly important because these patients have a higher incidence of IFD occurrence and a shorter incubation period. Different from the traditional view, for the allo-HSCT patients with PAP, itraconazole was a less effective treatment than other antifungal drugs. *Aspergillus spp*. represents the most common type of fungal infection in patients with allo-HSCT. Due to the limited diagnostic techniques, it is necessary to give antifungal therapy to patients who do not meet the EORTC/MSG 2008 criteria but show clinical evidence of fungal infection.

## Abbreviations

allo-HSCT: Allo-genetic hematopoietic stem cell transplantation
CAESAR: China Assessment of Antifungal Therapy in Hematological Disease
ELISA: Enzyme-linked immunosorbent assay
EORTC/MSG: European Organization for Research and Treatment of Cancer/Invasive Fungal Infections Cooperative Group and the National Institute of Allergy and Infectious Diseases Mycoses Study Group
HLA: Human leukocyte antigen
IFDs: Invasive fungal diseases
LFDs: Lateral flow devices
MALDI-TOF MS: Matrix-assisted laser desorption ionization TOF mass spectrometry
OS: Overall survival
PAP: Primary antifungal prophylaxis
SAP: Secondary antifungal prophylaxis.
